# Potent Inhibition of HIV-1 Replication by a Tat Mutant

**DOI:** 10.1371/journal.pone.0007769

**Published:** 2009-11-10

**Authors:** Luke W. Meredith, Haran Sivakumaran, Lee Major, Andreas Suhrbier, David Harrich

**Affiliations:** 1 Division of Infectious Diseases, Queensland Institute of Medical Research, Herston, Queensland, Australia; 2 Division of Immunology, Queensland Institute of Medical Research, Herston, Queensland, Australia; 3 School of Population Health, The University of Queensland, Herston, Queensland, Australia; 4 Griffith Medical Research College, Brisbane, Queensland, Australia; Tsinghua University, China

## Abstract

Herein we describe a mutant of the two-exon HIV-1 Tat protein, termed Nullbasic, that potently inhibits multiple steps of the HIV-1 replication cycle. Nullbasic was created by replacing the entire arginine-rich basic domain of wild type Tat with glycine/alanine residues. Like similarly mutated one-exon Tat mutants, Nullbasic exhibited transdominant negative effects on Tat-dependent transactivation. However, unlike previously reported mutants, we discovered that Nullbasic also strongly suppressed the expression of unspliced and singly-spliced viral mRNA, an activity likely caused by redistribution and thus functional inhibition of HIV-1 Rev. Furthermore, HIV-1 virion particles produced by cells expressing Nullbasic had severely reduced infectivity, a defect attributable to a reduced ability of the virions to undergo reverse transcription. Combination of these inhibitory effects on transactivation, Rev-dependent mRNA transport and reverse transcription meant that permissive cells constitutively expressing Nullbasic were highly resistant to a spreading infection by HIV-1. Nullbasic and its activities thus provide potential insights into the development of potent antiviral therapeutics that target multiple stages of HIV-1 infection.

## Introduction

Tat is an essential HIV-1 regulatory protein whose best described role is to promote high levels of viral gene expression via interactions with the HIV-1 transactivation response element (TAR) RNA [1], [2]. Full-length Tat is encoded by two exons comprising 101 amino acids (varying between 99 and 104 residues) and represents the most abundant form of Tat from patient-derived HIV-1. The first exon is organized into two major domains: the activation domain, which interacts with numerous cellular proteins including cyclin T1, and the basic domain, which is primarily comprised of arginine and lysine residues. The basic domain (amino acids 49–57) is required for many of Tat's activities including nuclear localization [3], [4] and TAR binding [5]. The basic domain has also been reported to facilitate other Tat activities such as membrane transduction [6], assisting HIV-1 reverse transcription [7] and augmenting integrin receptor binding [8].

A transdominant negative mutant is typically an altered form of a protein that can inhibit the normal function of its wild type counterpart. Engineered Tat proteins with altered basic domains possess transdominant negative phenotypes against wild type Tat. However, most studies of Tat transdominance have used one-exon *tat* mutants encoding truncated proteins of 72 amino acids or less. For example, Tat truncated at residue 53 can suppress transactivation initiated by wild type Tat [9]. This is despite the mutant localizing mainly to the cytoplasm of the cell, in contrast to wild type Tat, which localizes to the nucleus. One exon *tat* mutants with a deleted basic domain or where the basic domain has been substituted with neutrally-charged amino acids also recapitulate the transdominant negative effects on transactivation [10], [11]. Localization of Tat mutants to the nucleus, via fusion of the Tat nuclear localization signal to their carboxy termini, results in retention of the transdominant negative phenotype [10]. Moreover, mutations in the activation domain of the Tat mutant, which normally suppress the transactivation function of wild type Tat, can suppress transdominance [10].

The mechanism of transdominance of Tat basic-domain mutants is unclear. While several hypotheses have been proposed, studies suggest a model by which Tat mutants sequester one or more cofactors required for Tat-mediated transactivation [10], [12]. Sequestration of cofactors by transdominant Tat mutants may be mediated by an intact activation domain, but due to the disrupted basic domain, Tat mutants may be incapable of recruiting the cofactors (e.g., cyclin T1) to the site of transactivation [5], [13].

Here we describe for the first time the transdominant effects of a two-exon, 101 amino-acid Tat mutant called Nullbasic, which has the entire basic domain (amino acids 49–57) replaced with glycine/alanine residues. Surprisingly, the Nullbasic Tat mutant potently inhibited three different steps in HIV-1 replication. As expected, Nullbasic inhibited Tat-mediated transactivation. Unexpectedly, Nullbasic also potently inhibited Rev-mediated transport of HIV-1 mRNA. Furthermore, HIV-1 produced by cells expressing Nullbasic had greatly reduced infectivity due to a potent decrease in its ability to undergo reverse transcription. These combined activities meant that a cell line expressing Nullbasic was almost completely protected from high-dose HIV-1 challenge. These studies potentially highlight hitherto unrecognised activities of Tat and may open new avenues for therapeutic intervention.

## Results

### Nullbasic Is a Transdominant Tat Mutant

**Figure 1:**
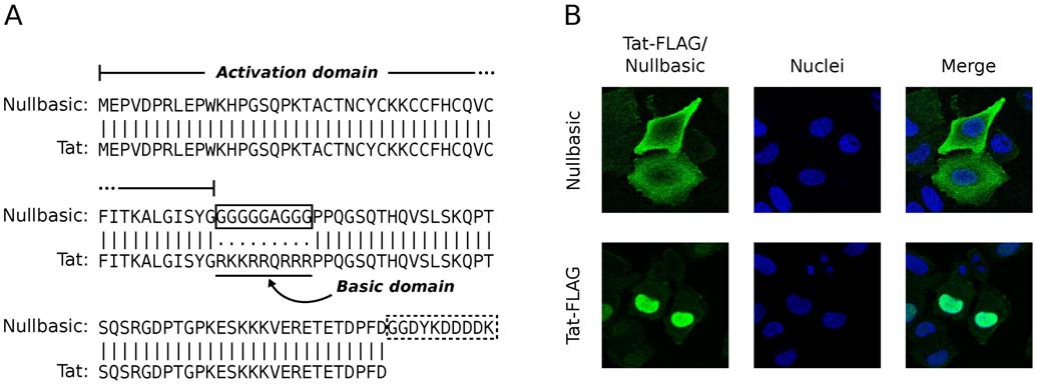
Nullbasic is a HIV-1 Tat mutant that localizes to the cell cytoplasm. (**A**) Amino-acid sequence alignment of Nullbasic (upper rows) against the BH10 clone of Tat (lower rows). Vertical bars indicate amino acid identity and dots indicate engineered substitutions. The solid box highlights the engineered basic domain mutations in Nullbasic, while the dashed box indicates a FLAG epitope tag added to the carboxy terminal. (**B**) HeLa cells expressing Nullbasic (top row) or Tat-FLAG (botton row) were visualized by confocal microscopy using anti-FLAG/FITC antibodies. Nuclei were stained with DAPI. Images are representative of 6 fields per slide from two independent experiments.

To investigate the molecular effects of transdominant Tat mutants, a novel mutant termed Nullbasic was created. Unlike previous studies, we mutated the full length, 101 amino-acid form of Tat since we noted that two-exon Tat is expressed at greater levels that one-exon Tat in most cell lines (unpublished observations), and that two-exon Tat is the primary form expressed by HIV-1 clinical isolates [14]. Nullbasic was engineered from the BH10 clone of Tat (101 amino acid variant) by replacing the basic domain with a glycine/alanine sequence and fusing the FLAG epitope tag to the carboxy terminus (Fig. 1A). One-exon transdominant Tat mutants with altered or deleted basic domains have previously been shown to localize predominantly to the cytoplasm of cells [3], [9], [10]. To see if Nullbasic similarly localizes to the cytoplasm, HeLa cells expressing Nullbasic or the wild type Tat-FLAG fusion protein were visualized by indirect immunofluorescence microscopy using anti-FLAG antibody. As expected, wild type Tat showed strong nuclear staining, whereas Nullbasic (like the one-exon transdominant Tat mutants) was mainly localized in the cytoplasm (Fig. 1B).

**Figure 2:**
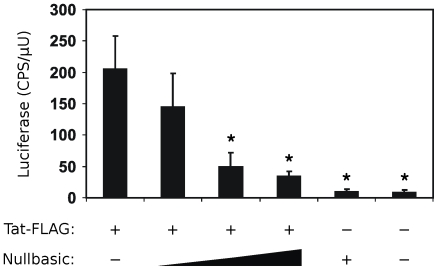
Nullbasic inhibits Tat-mediated transactivation. Increasing amounts (200 ng, 2 µg and 4 µg) or Nullbasic expression vector were titrated into cells co-transfected with constant amounts of Tat-FLAG plasmid, a luciferase reporter of Tat transactivation (HIV-1 LTR promoter) and a constitutive β-galactosidase expression plasmid (CMV promoter). Luciferase values (CPS, counts per second) were normalized to β-galactosidase activity (µU). Asterisks indicate significant decreases (*p*<0.05) in transactivation compared to uninhibited Tat-FLAG (first column). Columns represent the means and standard deviations of three independent experiments.

The defining feature of one-exon transdominant Tat mutants is their ability to suppress transactivation by wild type Tat [9], [10], [11], [15], [16]. To determine whether Nullbasic had similar activity, HeLa cells were co-transfected with equal amounts of plasmid encoding wild type Tat-FLAG, a luciferase-based HIV LTR transactivation reporter, a constitutive β-galactosidase expression vector and increasing amounts of plasmid encoding Nullbasic. The molar ratios of Tat-FLAG to Nullbasic plasmids assayed were 1∶1, 1∶10 and 1∶20. At the 1∶10 and 1∶20 ratios (Fig. 2, lanes 3 and 4, respectively), Nullbasic significantly suppressed transactivated luciferase expression compared to Tat transactivation in the absence of Nullbasic (lane 1). This effect was specific to Tat since Nullbasic did not significantly affect the CMV promoter-driven expression of β-galactosidase (data not shown). Thus Nullbasic is a transdominant inhibitor of Tat-mediated transactivation.

### Expression of Nullbasic in Cells Inhibits HIV-1 Production

**Figure 3:**
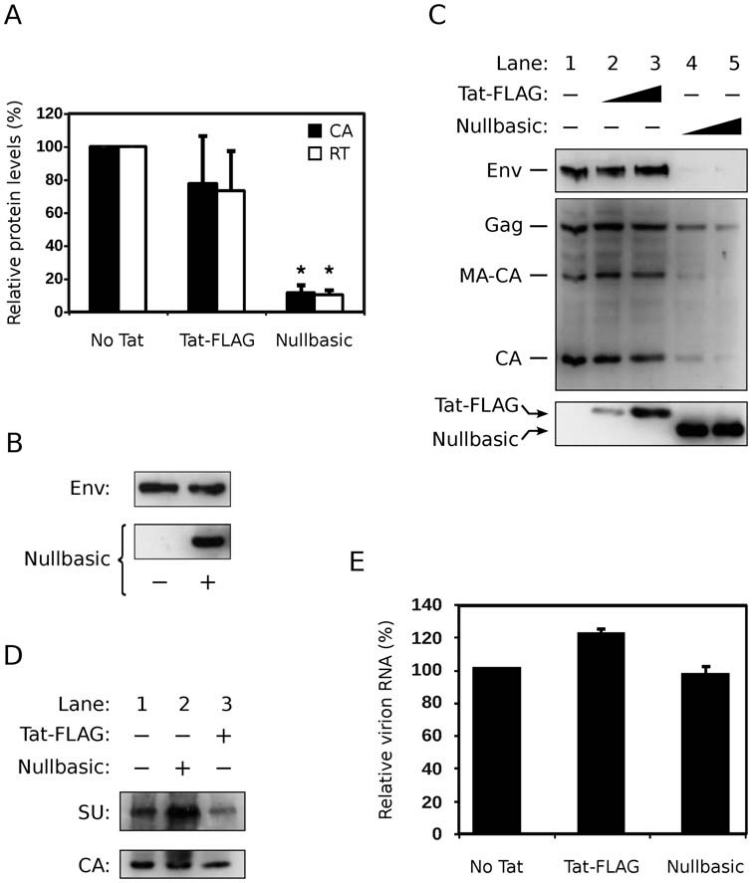
Nullbasic inhibits HIV-1 virion production but not composition. (**A**) Virions produced in HEK293T cells co-transfected with 1∶4 molar ratios of HIV-1 plasmid (pGCH) to Tat-FLAG, Nullbasic or empty vector (pcDNA3.1+; “No Tat”) plasmids were assayed for capsid (CA) and reverse transcriptase (RT) concentrations. The CA (black columns) and RT (white columns) concentrations are shown. Columns represent the means and standard deviations of four independent experiments and are expressed as a percentage of the “No Tat” sample. Asterisks indicate significant differences (*p*<0.025, Welch's *t*-test) in CA and RT concentrations between Nullbasic and Tat-FLAG samples. (**B**) HEK293T cells transfected with a reporter plasmid that expresses Rev-independent Env from the CMV/LTR hybrid promoter of pGCH were co-expressed with (lane 2) or without (lane 1) Nullbasic. Env and Nullbasic were detected by immunoblotting with anti-gp120 and anti-FLAG antibodies, respectively. (**C**) Cell lysates from HEK293T cells expressing pGCH provirus alone (lane 1) or co-expressing either Tat-FLAG (lanes 2 and 3; 1∶2 and 1∶4 molar ratios, respectively) or Nullbasic (lanes 4 and 5; 1∶2 and 1∶4 molar ratios, respectively) were immunoblotted with a monoclonal antibody against HIV-1 SU (upper panel), anti-serum against HIV (middle panel) and a monoclonal antibody against FLAG (lower panel). A β-galactosidase expression plasmid was included in all transfections and western blotting was performed on lysates normalised for β-galactosidase activity. (**D**) Culture supernatants were collected from HEK293T cells expressing pGCH alone (lane 1) or co-expressing either Nullbasic (lane 2) or Tat-FLAG (lane 3). Virions in the supernatants were concentrated by ultracentrifugation before samples containing 50 ng of total CA were immunoblotted with anti-gp120 and anti-CA antibodies. Data in B, C and D are representative of three independent experiments. (**E**) Packaged genomic RNA was isolated from virions produced by HEK293T cells expressing pGCH co-transfected with empty vector (pcDNA3.1+; “No Tat”) or co-expressing Tat-FLAG or Nullbasic before being quantitated by RT-PCR. The means and standard deviations of two experiments performed in duplicate on independent virus stocks are shown, with values expressed as a percentage of the “No Tat” sample.

To determine whether Nullbasic affects virus production, Nullbasic was co-expressed with a modified HIV-1 proviral construct, pGCH, in which the 5′ LTR U3 region has been replaced by the CMV immediate-early promoter. The promoter is thus a hybrid of the CMV promoter and the R and U5 regions of the HIV-1 LTR. This construct enables Tat-independent HIV-1 gene expression due to the presence of the CMV promoter, thereby allowing the investigation of any transactivation-independent effects of Nullbasic on the viral replication cycle. Virions were generated in HEK293T cells co-transfected with 1∶4 molar ratios of pGCH provirus to wild type Tat-FLAG, Nullbasic or empty vector (“No Tat”) plasmids. Virion samples were then assayed for capsid (CA) and reverse transcriptase (RT) protein concentrations by ELISA and colorimetric enzyme assay, respectively. The concentration of CA was 10-fold lower in supernatants from cells expressing pGCH and Nullbasic compared to cells expressing pGCH and Tat-FLAG (Fig. 3A). The concentration of RT was similarly decreased, indicating that Nullbasic expression suppressed overall virus production. To discount transdominant effects of Nullbasic on the CMV/LTR hybrid promoter of pGCH, a reporter construct expressing Rev-independent HIV-1 envelope (Env) [17] from the CMV/LTR promoter was co-expressed with Nullbasic in HEK293T cells. Nullbasic had no effect on the expression of Env from this reporter (Fig. 3B), indicating that Nullbasic does not significantly affect expression from the CMV/LTR promoter. Taken together, these experiments suggest that Nullbasic substantially reduces virion production by a mechanism independent of the inhibition of Tat transactivation.

To further examine the production of HIV-1 viral proteins, western blotting was performed on β-galactosidase-equalized lysates of cells co-expressing pGCH provirus and either Nullbasic or Tat-FLAG. Figure 3C shows that provirus-expressed Gag levels were reduced in the presence of Nullbasic (lanes 4 and 5) compared to Tat-FLAG (lanes 2 and 3). Gag-related proteolytic products, specifically HIV-1 p41*^gag^* (MA-CA) and CA, were proportionally reduced. This is consistent with the reduction in virion CA and RT protein levels observed in the supernatants of cells co-expressing Nullbasic (Fig. 3A). Expression of HIV-1 Env in the lysates was also reduced in the presence of Nullbasic (Fig. 3C). The decrease in Env levels by Nullbasic appeared to be greater than the decrease in Gag levels, but this is likely due to differences in antibody sensitivities since subsequent western blot analyses of purified virions showed CA and envelope surface antigen (SU) levels to be proportional when HIV-1 was co-expressed with either Nullbasic or Tat-FLAG (Fig. 3D). Finally, co-expression of Nullbasic did not significantly affect packaging of viral genomic RNA into HIV-1 virions compared to co-expression of Tat-FLAG (Fig. 3E). These experiments demonstrate that Nullbasic potently inhibits HIV-1 gene expression, leading to decreased production of the viral structural proteins without altering the relative protein or RNA compositions of virions.

### Nullbasic Downregulates Unspliced and Singly-Spliced HIV-1 mRNA Levels in Cells

**Figure 4:**
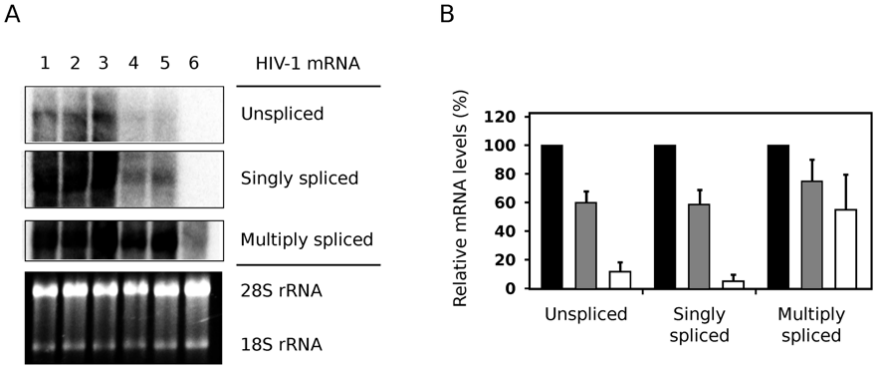
Nullbasic downregulates the levels of unspliced and singly-spliced HIV-1 mRNA expressed in cells. (**A**) Northern blot analysis of total RNA from HEK293T cells expressing pGCH provirus co-transfected with empty vector (pcDNA3.1+; “No Tat”, lane 1) or co-expressing increasing amounts of Tat-FLAG (lanes 2 and 3; 1∶2 and 1∶4 molar ratios, respectively) or Nullbasic (lanes 4 and 5; 1∶2 and 1∶4 molar ratios, respectively). An untransfected control was also included (lane 6). Unspliced, singly-spliced and multiply-spliced HIV-1 mRNA were detected with a single HIV-1-specific probe. 28S and 18S ribosomal RNA (rRNA) species demonstrate equal sample loading. Data are representative of four independent experiments. (**B**) Total RNA was extracted from HEK293T cells expressing pGCH co-transfected with empty vector (black bars) or co-expressing Tat-FLAG (gray bars) or Nullbasic (white bars) before RT-PCR reactions were performed using primers specific to unspliced, singly-spliced and multiply-spliced viral mRNA. Tat-FLAG and Nullbasic plasmids were transfected at 2∶1 molar ratios with respect to pGCH. The means and standard deviations of duplicate assays in three independent experiments are shown, with values for each mRNA class expressed as a percentage of the pGCH alone sample.

Expression of HIV-1 Gag and Env require nuclear export of unspliced and singly-spliced mRNA, respectively, by the viral Rev protein. Since transcription from pGCH is Tat independent, the observed downregulation of Env and Gag protein levels in Figure 3C could thus be due to Nullbasic interfering with HIV-1 mRNA nuclear export. To test this possibility, northern blot analysis was performed to determine the relative proportions of HIV-1 mRNA species produced in the presence of Nullbasic. HIV-1 mRNAs isolated from HEK293T cells co-expressing pGCH provirus and either Tat-FLAG or Nullbasic were detected with a single probe capable of binding to the unspliced, singly-spliced and multiply-spliced classes of HIV-1 transcripts. Figure 4A shows that both unspliced and singly-spliced mRNA had reduced intensities in the presence of Nullbasic (lanes 4 and 5) compared to an empty vector control (lane 1) or wild type Tat-FLAG (lanes 2 and 3). In contrast, multiply-spliced transcript levels were relatively unaffected by Nullbasic (compare lanes 4 and 5 to lanes 1 to 3). To confirm these results, quantitative RT-PCR was used to measure the effect of Nullbasic on viral mRNA levels (Fig. 4B). Compared to viral mRNA levels in the absence of overexpressed Tat, Tat-FLAG caused a modest decrease in all mRNA classes. Strikingly, however, Nullbasic induced strong suppression of unspliced and singly-spliced transcripts with little effect on multiply-spliced mRNA, in close agreement with the northern blot analysis (Fig. 4A). Nullbasic therefore reduces the steady state levels of Rev-dependent viral mRNA.

### Nullbasic Alters Rev Subcellular Localization and Inhibits Proviral Rev Function

**Figure 5:**
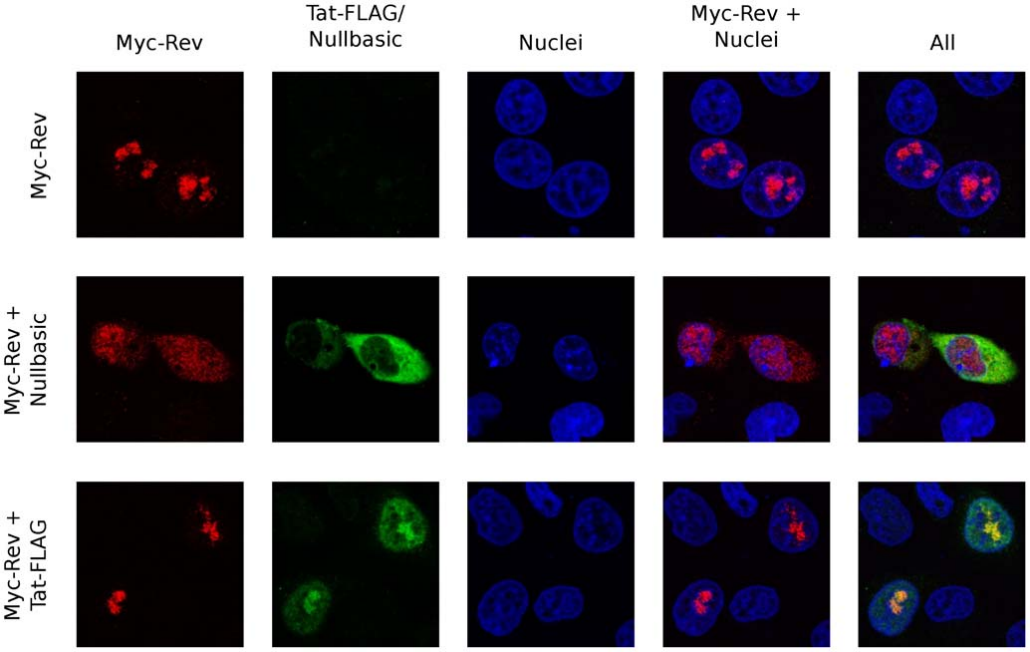
Nullbasic alters the subcellular localization of HIV-1 Rev. HeLa cells expressing a Myc-Rev fusion protein alone (top row), Myc-Rev with Nullbasic (middle row) or Myc-Rev with Tat-FLAG (bottom row) were visualized by confocal microscopy using anti-Myc/Cy3 and anti-FLAG/FITC antibodies. Nuclei were stained with DAPI. The total amounts of transfected plasmids in each experiment were normalized with empty vector (pcDNA3.1+). Images are representative of at least five fields per slide from three independent experiments.

HIV-1 Rev promotes the nuclear export of unspliced and singly-spliced viral mRNA by directly binding to the Rev response element (RRE) contained within these viral mRNAs. In the absence of Rev, multiple splicing events lead to the removal of sequences encoding Gag and Env. Rev∶mRNA complexes traffic from the nucleus to the cytoplasm via the CRM1 export pathway [18]. Rev traffics between the nucleus, nucleolus and cytoplasm to execute its mRNA export function, and previous reports implied that Tat and Rev share common trafficking mechanisms [21], [22]. Nullbasic could therefore interfere with Rev localization. To test this hypothesis, indirect immunofluorescence was performed on HeLa cells co-expressing Myc-Rev (a fusion between the MYC epitope tag and HIV-1 Rev) and either Nullbasic or Tat-FLAG using anti-MYC/Cy3 and anti-FLAG/FITC antibodies. Myc-Rev expressed alone accumulated in nuclear structures consistent with nucleoli, as observed previously [23] (Fig. 5). Co-expression with Nullbasic, however, caused substantial redistribution of Myc-Rev to the nucleoplasm and cytoplasm (Fig. 5, row 2). In contrast, co-expression with Tat-FLAG did not change Myc-Rev nucleolar localization (Fig. 5, row 3). These patterns of Myc-Rev distribution were frequently observed throughout the entire slide and were confirmed in two independent experiments. The results therefore illustrate that Nullbasic can alter the subcellular localization of Rev. However, immunoprecipitation analyses showed no substantial interaction between Myc-Rev and Nullbasic (data not shown). Together these data indicate that Nullbasic can alter Rev subcellular localization by an undetermined mechanism.

**Figure 6:**
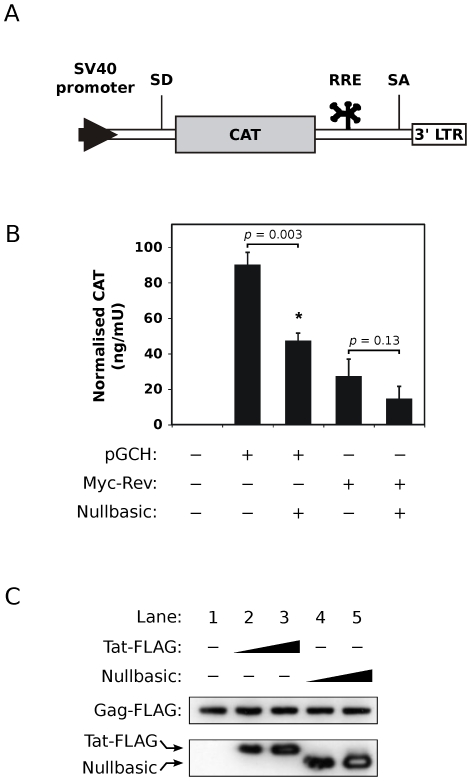
Nullbasic inhibits the RNA export function of provirus-expressed Rev. (**A**) Schematic diagram of the Rev-dependent CAT expression cassette in plasmid pDM128. The chloramphenicol acetyltransferase (CAT) gene exists within an artificial intron bounded by splice donor (SD) and splice acceptor (SA) sequences. Successful expression of CAT protein requires binding of Rev to the Rev response element (RRE) to avoid intron splicing and to enable nuclear export of the CAT mRNA transcript. SV40: simian virus 40; 3′ LTR: HIV-1 long terminal repeat (contains polyadenylation signal). (**B**) HEK293T cells were co-transfected with pDM128 and pGCH provirus alone (column 2), Myc-Rev plasmid alone (column 4) or along with Nullbasic plasmid (columns 3 and 5). Empty vector (pcDNA3.1+) was used to normalize the total amount of transfected plasmids. A pDM128 only transfection was included as a negative control (column 1) and a constitutive β-galactosidase plasmid was included in all transfections to account for variations in transfection efficiencies. CAT expression was measured by ELISA and is expressed relative to β-galactosidase activity (ng/mU). Columns represent means and standard deviations of three independent experiments. (**C**) Lysates from cells expressing a Rev-independent Gag-FLAG fusion protein co-transfected with empty vector (lane 1) or co-expressing either Tat-FLAG (lanes 2 and 3; 1∶2 and 1∶4 molar ratio, respectively) or Nullbasic (lanes 4 and 5; 1∶2 and 1∶4 molar ratio, respectively) were immunoblotted with anti-FLAG antibody to detect all three proteins. The white band centers in lanes 3 and 5 are due to luminol precipitation, indicative of very high amounts of Tat-FLAG and Nullbasic protein, respectively, on the membrane. Data are representative of three independent experiments.

To assess whether Nullbasic could downregulate the mRNA export function of Rev, a Rev reporter assay was used that makes use of the pDM128 plasmid (Fig. 6A) [19]. In the absence of Rev, the host-cell splicing machinery removes the chloramphenicol acetyltransferase (CAT) open reading frame from mRNA transcripts expressed by the plasmid's SV40 promoter (Fig. 6A), resulting in no production of CAT protein. In the presence of Rev, the splicing machinery is bypassed when Rev interacts with the RRE element present in pDM128 transcripts, thereby allowing CAT protein expression. When the pGCH provirus was used to provide Rev in the reporter assay, co-expression of Nullbasic resulted in a statistically-significant decrease (*p* = 0.003) of CAT protein levels compared with no Nullbasic co-expression (Fig. 6B). When we used the Myc-Rev expression plasmid to provide Rev in the assay, Nullbasic appeared to inhibit CAT protein levels but this was determined not to be significant (Fig. 6B; *p* = 0.13, compared to no Nullbasic co-expression). Nullbasic alone did not induce CAT expression and downregulation of Rev function was not caused by diminished proviral Rev steady state (data not shown). The results therefore suggest that Nullbasic can inhibit proviral Rev-mediated mRNA export, possibly requiring the presence of other viral factors to induce this inhibition.

To provide further evidence that Nullbasic inhibits Rev-mediated mRNA export function, a Gag-FLAG fusion protein expressed from a plasmid in which inhibitory sequences had been silenced to allow Rev-independent Gag production [20] was co-expressed with Nullbasic. In contrast to wild type Gag (Fig. 3C), the steady state levels of Rev-independent Gag-FLAG were the same in the presence of both Tat-FLAG and Nullbasic (Fig. 6C), indicating that Nullbasic had no effect on Gag expression or protein levels when the requirement for Rev was bypassed. Together the data therefore suggest that Nullbasic interferes with the mRNA export function of HIV-1 Rev.

### Nullbasic Inhibits HIV-1 Infectivity and Reverse Transcription

**Figure 7:**
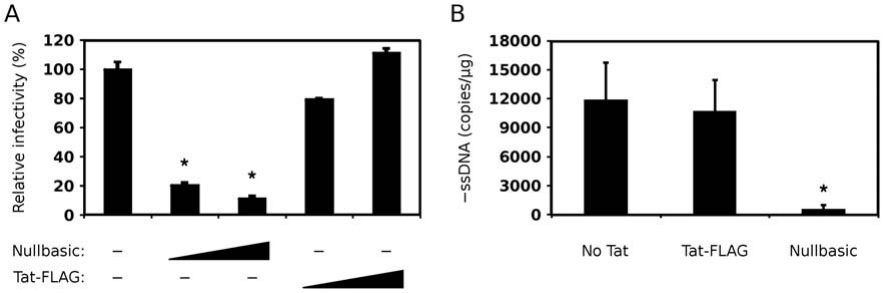
Nullbasic inhibits HIV-1 infectivity and endogenous reverse transcription. (**A**) The infectivity of HIV-1 produced by HEK293T cells expressing pGCH provirus co-transfected with empty vector (pcDNA3.1+) or co-expressing increasing amounts of Nullbasic or Tat-FLAG (at 1∶2 and 1∶4 molar ratios) were determined by the MAGI assay. The indicator cells were infected with virion samples equalized for reverse transcriptase activity before cell lysates were assayed for β-galactosidase production 48 h later. Columns represent the means and standard deviations of three independent experiments and are expressed as a percentage of the “No Tat” sample (column 1). Asterisks indicate significant differences (*p*<0.05) in infectivities compared to the “No Tat” sample. (**B**) Detergent-free endogenous reverse transcription assays were performed with the same virus samples as in A. The amount of negative-strand strong-stop DNA (–ssDNA) was quantitated by PCR, and normalized to the RT activity in each sample. The asterisk indicates a significant difference (*p*<0.05) in –ssDNA synthesis relative to the “No Tat” sample, and columns represent the means and standard deviations of duplicate assays in three independent experiments.

We next compared the infectivity of virions produced in the presence and absence of Nullbasic. Infectivity was tested using HeLa-CD4-LTR-β-gal reporter (MAGI) cells, which contain a stably-integrated LTR-β-galactosidase expression cassette that reports productive HIV-1 infection following Tat expression and transactivation [24]. Virion samples tested in the assay were always normalized for RT activity. We observed that HIV-1 virions produced by Nullbasic-expressing cells had significantly reduced infectivities compared to virons produced by Tat-FLAG-expressing cells (Fig. 7A). This was evident when the molar ratio of pGCH to Nullbasic plasmid was 1∶2 and 1∶4. Nullbasic therefore reduces HIV-1 infectivity when expressed in the virus producer cells.

A detergent-free endogenous reverse transcription assay was used to determine whether virions produced by Nullbasic-expressing cells were able to initiate reverse transcription [25]. There was an 18-fold decrease in minus-strand strong-stop DNA (–ssDNA) synthesis, an early product of reverse transcription, in virions produced in the presence of Nullbasic compared to virions produced in the presence of Tat-FLAG (Fig. 7B). These data suggest that the suppression of virion infectivity was primarily due to a Nullbasic-induced defect in reverse transcription.

### HIV-1 Replication Is Potently Inhibited in a Permissive Cell Line Stably Expressing Nullbasic

**Figure 8:**
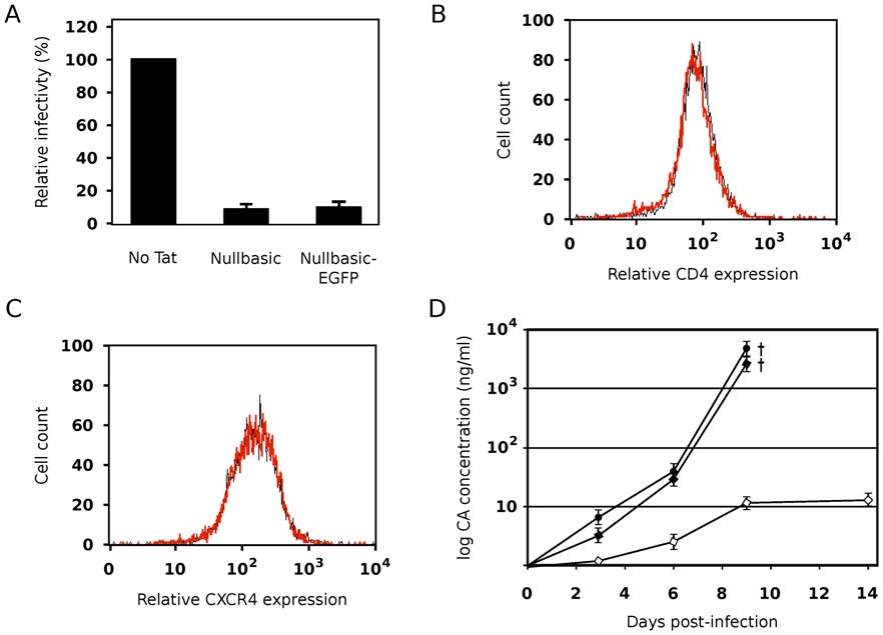
Expression of Nullbasic-EGFP in permissive target cells suppresses HIV-1 infection and spread. (**A**) The ability of Nullbasic-EGFP to inhibit HIV-1 infectivity was compared to Nullbasic in a MAGI assay similar to Figure 7A (using a 1∶2 molar ratio only). Columns represent the means and standard deviations of three independent experiments and are expressed as a percentage of the “No Tat” (pcDNA3.1+ co-transfected) sample. (**B**) Cell surface expression of transgenic CD4 receptors was quantitated in MAGI (black) and MAGI/Nullbasic-EGFP (red) cells by flow cytometry using an anti-CD4 monoclonal antibody. (**C**) Cell surface expression of endogenous CXCR4 receptors was quantitated in MAGI (black) and MAGI/Nullbasic-EGFP (red) cells by flow cytometry using an anti-CXCR4 monoclonal antibody. (**D**) The MAGI (black circles), MAGI/EGFP (black diamonds) and MAGI/Nullbasic-EGFP (white diamonds) cell lines were infected with equal amounts of pGCH-derived virions (500 ng CA-equivalent per 10^6^ cells) before viral replication was monitored over a 14-day period. Log values of virion CA levels measured in the culture supernatants are shown plotted against time. Obelisks (†) indicate observations of syncytia and cell death in the MAGI and MAGI/EGFP cell lines at 9 days post-infection. Data points represent the means and standard deviations of triplicate assays in two independent experiments.

We next investigated if expression of Nullbasic in permissive target cells can confer resistance to HIV-1 infection. To enable convenient monitoring of Nullbasic expression, the enhanced green fluorescent protein (EGFP) was fused to the carboxy terminal of Nullbasic. Fusion of EGFP to Nullbasic did not alter its antiviral activity compared to unfused Nullbasic (Fig. 8A). Nullbasic-EGFP was stably introduced into MAGI cells via lentivirus-mediated transduction [26], and highly expressing cells were isolated by fluorescence-activated cell sorting (FACS). A control cell line expressing only EGFP (MAGI/EGFP) was similarly derived. Stable expression of Nullbasic-EGFP and EGFP was retained in the respective cell lines after several passages, as demonstrated by both fluorescence microscopy and western blotting (data not shown). Lentiviral transduction was determined by flow cytometry not to alter either transgenic CD4 (Fig. 8B) or endogenous CXCR4 (Fig. 8C) receptor levels in the MAGI/Nullbasic-EGFP cell line.

The parental and transduced cell lines were challenged with equal amounts of HIV-1 virions (500 ng CA-equivalent per 10^6^ cells) and cultured for 14 days. Virus production from the cells was monitored over time by CA ELISA of the culture supernatants. A rapid increase in virus production in both the parental MAGI and MAGI/EGFP cell lines was seen over the first 9 days before syncytia formation and cell death were observed (Fig. 8D). In contrast, a greater than two-log reduction in virus production was observed in the MAGI/Nullbasic-EGFP cell line, with no syncytia formation or cell death being apparent even at the end of the experiment. These results were confirmed in two independently derived MAGI/Nullbasic-EGFP cell lines. Expression of Nullbasic in permissive cells is therefore protective against HIV-1 challenge and suppresses viral spread.

## Discussion

Transdominant-negative Tat mutants have to date been mainly defined by their abilities to suppress the transactivation function of HIV-1 Tat [9], [10], [11], [15], [16] and to induce latency during HIV-1 infection [27], [28]. Here we describe for the first time a full-length, transdominant two-exon Tat mutant, termed Nullbasic (Fig. 1A), and demonstrate its potent inhibitory activity against multiple stages of the HIV-1 replication cycle. In addition to suppressing Tat transactivation (Fig. 2), Nullbasic reduced HIV-1 virion production by suppressing Rev-dependant RNA export (Figs. 3 and 4) and virus produced in the presence of Nullbasic was severely defective for reverse transcription (Fig. 7B). As a result of these multiple inhibitory activities, expression of Nullbasic in permissive cells conferred strong resistance against high-dose HIV-1 challenge and the reduction by greater than two orders of magnitude of a spreading viral infection (Fig. 8). To our knowledge, this is the first demonstration of a transdominant Tat mutant that targets multiple, distinct steps in the HIV-1 replication cycle: proviral gene transcription, Rev-dependent mRNA transport and reverse transcription.

Nullbasic downregulated the expression of Gag and Env from a HIV-1 proviral plasmid that expressed viral mRNA from a CMV promoter (Fig. 3C). Two different assays, quantitation of viral mRNA (Fig. 4) and a functional assay (Fig. 6), pointed to a defect in Rev mRNA export function. Nullbasic appeared to disrupt Rev distribution (Fig. 5) and function leading to decreased steady state levels of unspliced and singly-spliced viral mRNA, resulting in the observed downregulation of Gag and Env protein levels, respectively. Interestingly, Nullbasic did not significantly affect the mRNA export function of ectopically-expressed Rev (Fig. 6B, Myc-Rev), suggesting that a complex interaction between Nullbasic, Rev and other HIV-1 factors is required to inhibit viral mRNA export. Furthermore, the data imply that Nullbasic inhibits Rev function via an indirect mechanism. Further investigation is required to determine which viral or cellular factor intermediates between Nullbasic and Rev to enable viral mRNA export inhibition.

Rev normally binds the Rev response element (RRE), an RNA structure located within HIV-1 *env*, to facilitate export of unspliced and singly-spliced mRNA from the nucleus. A dominant-negative Rev mutant called M10 has been described that was shown to inhibit wild type Rev function [29]. The M10 mutant protein is dominant negative because it retains the ability to bind the HIV-1 RRE but is unable to promote export of the viral mRNA from the nucleus, thereby inhibiting HIV-1 replication [29], [30]. Our data suggest that Nullbasic and M10 inhibit Rev by different mechanisms. Confocal microscopy experiments indicated that Nullbasic can disrupt Rev subcellular localization (Fig. 5). The nucleolar localization of Rev appears to be important for its function [23], [31], so the Nullbasic-induced redistribution of Rev from nucleolus to nucleoplasm and cytoplasm is likely to be necessary, but is not sufficient, to inhibit Rev function. There are reports that Tat and Rev share common nuclear trafficking pathways involving importin β [22] and B23 (nucleophosmin) [21], [23], so it is possible that Nullbasic may directly interfere with Rev trafficking. Tat trafficking, however, remains controversial, with conflicting reports that Tat nuclear accumulation requires active, factor-dependent pathways [32] or passive, factor-independent mechanisms [33]. The lack of a demonstrable interaction between Nullbasic and Myc-Rev in immunoprecipitation experiments (unpublished observations) suggests that Nullbasic interferes with Rev trafficking by an indirect mechanism. Conceivably, Nullbasic may sequester cellular factors (such as importins) normally required for Rev nucleolar targeting. Whatever the mechanism, disruption of Rev trafficking and therefore HIV-1 mRNA export function represents a major antiviral activity of Nullbasic.

The third major activity of Nullbasic was abrogation of intravirion reverse transcription activity (Fig. 7B). Co-expression of Nullbasic in virus producer cells did not alter the RNA content of virions (Fig. 3E), indicating that Nullbasic does not affect HIV-1 genomic RNA packaging. Tat is usually only present in virions at very low concentrations [34]. However, increased nonspecific inclusion of Nullbasic into virions may occur due to the high cytoplasmic concentrations of Nullbasic (Fig. 1B). Once in the virion, Nullbasic may have a dominant negative effect on Tat-mediated enhancement of reverse transcription [25]. Alternatively, Nullbasic may negatively affect nucleocapsid activity, which was recently reported to precisely regulate reverse transcription [35]. Nullbasic might also bind other crucial intravirion factors and thereby indirectly disrupt the regulation of reverse transcription. Whatever the mechanism, virions produced by cells expressing Nullbasic have low infectivity most likely due to a reverse transcription defect.

The potential therapeutic value of Nullbasic is illustrated by experiments showing that Nullbasic expression protected cells from high-dose HIV-1 infection (500 ng CA-equivalent virions per 10^6^ cells) by greater than two orders of magnitude compared to control cells (Fig. 8D). Furthermore, Nullbasic-expressing cells were protected against HIV-1-induced syncytia formation and cell death, indicating that Nullbasic expression protected cells against a spreading infection. All three of the previously described antiviral activities of Nullbasic (inhibition of transactivation, Rev function and reverse transcription) likely combined to suppress this spreading infection.

In conclusion, we demonstrate the potent antiviral activity of a transdominant Tat mutant and show that multiple steps in the HIV-1 replication cycle are targeted. In addition to negative effects on viral gene expression, we report for the first time that Nullbasic also inhibits Rev-dependent viral mRNA transport and intravirion reverse transcription. Moreover, these inhibitory effects combined potently to reduce HIV infection, illustrating Nullbasic and its activities as potential avenues for the development of new therapeutic interventions. Identification of the cellular or viral factors that interact with Nullbasic to induce Rev and reverse transcription inhibition may also reveal novel aspects of HIV-1 replication.

## Materials and Methods

### Cell Culture and Transfections

HeLa and HEK293T cells were cultured in RPMI 1640 medium supplemented with 100 U/ml penicillin, 100 µg/ml streptomycin and 10% (v/v) newborn bovine serum (Invitrogen Corporation). HeLa-CD4-LTR-β-gal (MAGI) cells [24] were obtained from Michael Emerman through the NIH AIDS Research and Reference Reagent Program, Division of AIDS, NIAID, NIH. The cells were maintained in the same medium as above but supplemented with 0.2 mg/ml G418 and 0.1 mg/ml hygromycin B. All cells were incubated at 37°C under a humidified atmosphere of 5% CO_2_ in air. Transfections were performed with Lipofectamine 2000 (Invitrogen) or FuGENE 6 (Roche Diagnostics Corporation) transfection reagents according to the manufacturers' instructions. Transfections were performed in 6-cm dishes for reporter assays and western blotting, and 10-cm dishes for HIV-1 virion production.

### Plasmids

The plasmid expressing the two-exon, 101 amino-acid, BH10 clone of Tat fused to the FLAG epitope (pcDNA3.1/Tat-FLAG) was a gift from Monsef Benkirane, Institut de Génétique Humaine, France. Nullbasic was created by firstly removing the basic domain sequence (corresponding to amino acids 49–57 in Tat) in pcDNA3.1/Tat-FLAG by inverse PCR before complementary oligonucleotides encoding the amino acid sequence, Gly–Gly–Gly–Gly–Gly–Ala–Gly–Gly–Gly were annealed and ligated to form pcDNA3.1/Nullbasic. Correct orientation of the insert was determined by DNA sequencing. To create the Nullbasic-EGFP-encoding lentivector pLOX-CW/Nullbasic-EGFP, the *EGFP* gene from pIRES2-EGFP (Clontech Laboratories) was cloned onto the 3′ end of Nullbasic before the Nullbasic-EGFP cassette was subcloned to replace *gfp* in pLOX-CWgfp [26] using *Bam* HI and *Sal* I restriction sites. The Tat-transactivation luciferase reporter pGL3-LTR consists of the long terminal repeat from HIV-1 clone SF2 cloned into pGL3-basic (Promega Corporation) via *Bam* HI and *Hind* III restriction enzyme sites. The LTR spans nucleotides −180 to +81, relative to the start of transcription. A Rev-independent Env expression construct, pNL1.5E-RTEm26CTE [17], was a gift from Barbara Felber, National Cancer Institute, Maryland, USA. The Env-RTEm26CTE open reading frame was subcloned to replace the HIV-1 genome in pGCH using *Bss* HI and *Xho* I restriction enzymes, thus forming pGCH-Env-RTEm26CTE. The Rev-independent Gag expression construct pCMV5-Gag [20] was a gift from Marilyn Resh and George Pavlakis, National Cancer Institute, Maryland, USA. The FLAG epitope sequence was added to the 3′ end of *gag* by inverse PCR mutagenesis. A plasmid expressing the BRU clone of Rev (pRSV-Rev) was a gift from Damian Purcell, University Melbourne, Australia. The MYC epitope sequence was added to the 5′ end of *rev* by inverse PCR mutagenesis before the Myc-Rev cassette was subcloned into pcDNA3.1+ (Invitrogen). The β-galactosidase expression plasmid pCMVβ [36] was used as a transfection control in various experiments as indicated. β-galactosidase activity was measured by the chlorophenol red-β-D-galactopyranoside (CPRG)-based assay [37]. The HIV-1 proviral expression vector pGCH will be described elsewhere (L. Meredith *et al.*, manuscript in preparation).

### Indirect Immunofluorescence

HeLa cells were grown on coverslips and transfected with plasmids as above. Cells were fixed 24 h later in 3% (w/v) paraformaldehyde, quenched with 50 mM NH_4_Cl, permeabilized with 0.1% (v/v) Triton X-100 and blocked in 10% (v/v) normal goat serum (Millipore Corporation). Tat-FLAG and Nullbasic were probed with mouse anti-FLAG M2 monoclonal antibody (Sigma-Aldrich Incorporated) and FITC-conjugated goat anti-mouse antibody (Invitrogen). Myc-Rev was probed with rabbit anti-MYC polyclonal antibody (Cell Signaling Technology Incorporated) and Cy3-conjugated goat anti-rabbit antibody (Invitrogen). Nuclei were stained with 1 µM 4′,6-diamidino-2-phenylindole (DAPI; Invitrogen) and coverslips were mounted onto slides with SlowFade Gold mounting medium (Invitrogen). Images were acquired with a Leica TCS SP2 confocal system (Leica Microsystems) using an oil-immersion 63× objective lens and standard lasers and filters for FITC, Cy3 and DAPI (two-photon) fluorescence.

### Transactivation Assay

HeLa cells were co-transfected with 200 ng of Tat-FLAG, 500 ng of pGL3-LTR, 300 ng of pCMVβ and either 200 ng, 2 µg or 4 µg of Nullbasic plasmid. Cells were harvested 24 h post-transfection and cell lysates prepared with phosphate-buffered saline (PBS) containing 0.5% (w/v) Triton X-100 and protease inhibitors (Roche). Lysates were assayed for luciferase activity using the Steady-Glo luciferase assay system (Promega). β-galactosidase activity was assayed as above.

### HIV-1 Virion Infectivity

For the effect of Nullbasic on virion infectivity experiment, HEK293T cells were transfected with 5 µg of pGCH provirus and either 4 µg or 8 µg of Nullbasic or Tat-FLAG plasmids. Supernatants were collected 48 h post-transfection, filtered through 0.45 µm filters and virion concentrations were determined by RT colorimetric assay (Roche). MAGI cells were infected with 20 ng RT-equivalent of virions for 2 h and allowed to incubate for a further 46 h. Cells were then lysed and assayed for β-galactosidase expression using the CPRG assay [37]. Total cellular protein amounts were measured using the Bradford assay [38], and was used to normalise β-galactosidase expression. For the effect of Nullbasic on viral CA and RT levels experiment, HEK293T cells were transfected with 5 µg of pGCH and either 4 µg or 8 µg of Tat-FLAG or Nullbasic plasmids. Viral supernatants were collected 48 h post-transfection, filtered, and CA and RT concentrations were determined by ELISA (Zeptometrix Corporation) and colorimetric enzyme assay (Roche), respectively.

### Western Blot

For the western blotting of cell lysates, HEK293T cells were transfected with either 5 µg of pGCH provirus, 1.5 µg of pGCH-Env-RTEm26CTE or 2 µg of pCMV5-Gag-FLAG. Cells were also co-transfected with 250 ng of pCMVβ and either Tat-FLAG or Nullbasic plasmids as indicated. Cells were lysed 24 h post-transfection and assayed for β-galactosidase and total protein concentrations as above. Lysates equivalent in β-galactosidase activity were boiled in sample buffer and electrophoresed in a sodium dodecylsulfate-containing polyacrylimide gel according to the methods of King and Laemmli [39]. Proteins were electroblotted to a polyvinylidene difluoride (PVDF) membrane (GE Healthcare) using a semi-dry transfer system (Bio-Rad). Tat-FLAG, Nullbasic and Gag-FLAG were detected with mouse anti-FLAG M2 monoclonal antibody (Sigma-Aldrich). HIV-1 Env and SU were detected with mouse anti-gp120 monoclonal antibody (a gift from Andy Poumbourios, Burnet Institute, Australia). Other HIV-1 proteins were detected with HIV-IG anti-serum (AIDS Research and Reference Reagent Program, Division of AIDS, NIAID, NIH). Mouse primary antibodies were detected with horseradish peroxidase (HRP)-conjugated goat anti-mouse antibody (Invitrogen), and HIV anti-serum was detected with HRP-conjugated goat anti-human IgG anti-serum (Sigma-Aldrich).

### Virion RNA Packaging Assay

HEK293T cells were co-transfected with 5 µg of pGCH provirus and 4 µg of either Tat-FLAG or Nullbasic plasmids. Culture supernatants were harvested and treated with DNase I to remove contaminating plasmid DNA before being ultracentrifuged through a 20% (v/v) sucrose cushion at 100 000×*g* for 2 h. Packaged RNA from the virion pellets were extracted with TRIzol reagent (Invitrogen) before being reverse transcribed with random hexamers and Superscript III MMLV RT (Invitrogen) according to the manufacturer's instructions. cDNA was measured by quantitative PCR with Platinum SYBR Green qPCR supermix (Invitrogen) on the Rotor-Gene 6000 (Corbett Life Science) using primers, 5′–TCT CTA GCA GTG GCG CCC GAA CAG GG and 5′–GTC GCC GCC CCT CGC CTC TTG. To control for reaction efficiency, kanamycin cassette control RNA (Promega) was added to the extracted RNA mixture and assayed as above with primers, 5′–GGC TCG CGA TAA TGT CGG G and 5′–GAT GGT CGG AAG AGG C. Quantitated kanamycin cDNA levels were used to normalise viral cDNA levels.

### Northern Blot and RNA Splicing Assay

HEK293T cells were transfected with 5 µg of pGCH provirus and 4 µg or 8 µg of either Tat-FLAG or Nullbasic plasmids. Total RNA was extracted 48 h post-transfection using TRIzol reagent (Invitrogen) according to the manufacturer's instructions. Twenty micrograms of RNA samples were electrophoresed in a 1% (w/v) agarose gel containing 0.6 M formaldehyde and either stained with ethidium bromide to visualize ribosomal RNA, or blotted to a nitrocellulose membrane using a TurboBlotter transfer system (Schleicher and Schuell). RNAs were cross-linked to the membrane with ultraviolet light and heat, and HIV-1 mRNA species were detected with a ^32^P-labelled probe corresponding to the *Bam* HI–*Xho* I fragment in the 3′ LTR of HIV-1. Hybridizations were visualized with a Typhoon 8600 imager (GE Healthcare). For the RNA splicing assay, total RNA obtained for the northern analysis was used as a template for quantitative RT-PCR as described above. The primers used to detect unspliced, singly-spliced and multiply-spliced viral mRNA have been previously described [40]. Kanamycin cassette control RNA, as described above, was included in the assay to normalise for reaction efficiency.

### Rev Reporter Assay

HEK293T cells were transfected with either 1 µg of pGCH or 20 ng of pcDNA3.1/Myc-Rev, along with 100 ng of pDM128, 100 ng of pCMVβ and 1.5 µg of either Nullbasic or empty vector (pcDNA3.1+) plasmids. Cells were harvested and lysed 24 h post-transfection before CAT expression was assayed by ELISA (Roche) according to the manufacturer's instructions. β-galactosidase activity was assayed as above.

### Establishment of the MAGI/Nullbasic-EGFP Cell Line

Pseudotyped lentivirus particles were generated by co-transfecting HEK293T cells with pLOX-CW/Nullbasic-EGFP or pLOX-CWgfp along with pCMVΔR8.91 [41] and pHEF-VSV-G (a gift from Sabine Piller, Westmead Millennium Institute, Australia). MAGI cells were transduced with lentivirus particles in the presence of hexadimethrine bromide (8 µg/ml; Sigma-Aldrich) for 48 h before transduction was confirmed by fluorescence microscopy. Highly expressing cells were isolated by FACS using a MoFlo cell sorter (Beckman Coulter Incorporated), and expression of Nullbasic was confirmed by western blot analysis.

### Flow Cytometry of Cell-Surface Receptor Levels

MAGI/Nullbasic-EGFP and nontransduced MAGI cells were incubated with mouse anti-CD4 or mouse anti-CXCR4 monoclonal antibodies (R&D Systems) followed by Cy5-conjugated goat anti-mouse antibody (Invitrogen). Receptor levels were quantitated by measuring Cy5 fluorescence using a FACScalibur flow cytometer (Becton Dickinson), counting 10^5^ cells per sample.

### Detergent-Free Endogenous Reverse Transcription Assay

HIV-1 virions from HEK293T cells co-transfected with 5 µg of pGCH provirus and 4 µg of either Tat-FLAG or Nullbasic plasmids were assayed for endogenous (intravirion) reverse transcription as previously described [42]. Virions were normalized for equivalent RT activity before assay. The primers used to quantitate minus-strand strong-stop DNA were 5′–GGG TCT CTC TGG TTG ACC AGA and 5′–ACA CAA CAG ACG GGC ACA CAC.

### Viral Replication Kinetics

MAGI/Nullbasic-EGFP, MAGI/EGFP and nontransduced MAGI cells were infected with high doses (500 ng CA-equivalent) of pGCH-derived HIV-1 for 2 h. Non-adsorbed virions were removed by washing cells with PBS before infected cells were incubated for a 14-day period. Culture supernatants were periodically sampled for virion production by CA ELISA in triplicate.

### Statistical Analyses

Hartley's *F*_max_ test was used to determine variance homoscedasticity between data sets. Student's *t*-test was used to evaluate null hypotheses for homoscedastic data, while Welch's *t*-test was used for heteroscedastic data. The underlying distributions were two tailed for all tests and significant difference was defined as *p*<0.05.
